# Precautions for Patients Taking Tamoxifen

**Published:** 2018-01-01

**Authors:** Mary Heery, Patrick Corbett, Richard Zelkowitz

**Affiliations:** Western Connecticut Health Network at The Smilow Family Breast Health Center, Norwalk Hospital, Norwalk, Connecticut

## Abstract

Tamoxifen is a lifesaving treatment for millions of breast cancer patients worldwide. Yet taking tamoxifen may be challenging for some patients due to issues of compliance, drug interactions, and surgical considerations. Educating patients with a one-page teaching sheet, "Precautions for Patients Taking Tamoxifen," may improve tamoxifen’s effectiveness and prevent complications. Advanced practitioners are in a position to prescribe tamoxifen, review medication interactions, educate patients, impact patients’ quality of life, improve patients’ sense of control, and increase patients’ partnerships with their oncology providers.

Tamoxifen is the most widely prescribed breast cancer therapy; millions of patients take tamoxifen worldwide. Tamoxifen is an oral medication that treats hormone-sensitive breast cancers. A hormone-sensitive breast cancer is estrogen receptor–positive (ER+), progesterone receptor–positive (PR+), or both. Approximately 70% of breast cancers are ER+ (Allred et al., 2012). Research has demonstrated that tamoxifen therapy reduces the annual breast cancer death rate by 31% (Early Breast Cancer Trialists’ Collaborative Group, 2005) and reduces the risk of developing a contralateral breast cancer by as much as 50% (Early Breast Cancer Trialists’ Collaborative Group, 1998).

## RESEARCH

Tamoxifen is a selective estrogen receptor modulator that blocks the transcriptional activity of estrogen receptors by directly binding to them, producing a nuclear complex that decreases DNA synthesis and inhibits estrogen effects (Ali, Rasool, Cahaoudry, & Jamal, 2016). Tamoxifen has been studied since the early 1970s after it failed as a postcoital contraceptive (Jordan, 2008). It is prescribed in multiple patient diagnoses. Tamoxifen is widely used as an adjuvant therapy in early-stage invasive breast cancer and ductal carcinoma in situ (Fisher et al., 1989). It has long been shown to work in advanced breast cancer disease. It is also the most effective treatment for men with breast cancer (Fentiman, Fourquet, & Hortobagyi, 2006). In the National Surgical Adjuvant Breast and Bowel Project’s Breast Cancer Prevention Trial, tamoxifen reduced the incidence of breast cancer in women who are at high risk for breast cancer (Fischer et al., 1998). It has long been known to be effective in postmenopausal women who are not able to tolerate aromatase inhibitors. Ongoing trials report that 10 years of tamoxifen is better than 5 years (Davises et al., 2013). Both the Adjuvant Tamoxifen: Longer Against Shorter (ATLAS) trial, which enrolled 6,846 women, and the adjuvant Tamoxifen—To offer more? (aTTom) trial, which enrolled 6,953 women, reinforce the evidence that more than 5 years of tamoxifen is beneficial (Azvolinsky, 2013). The combined data showed an additional 25% reduction in breast cancer mortality 10 years and beyond the diagnosis of breast cancer. In fact, reduced mortality from cancer was seen 10 to 14 years after a breast cancer diagnosis. Ongoing tamoxifen studies will continue to report findings with follow-up of thousands of women for many years to come.

Tamoxifen is the backbone for breast cancer treatment; however, it poses challenges for patients and providers. A daily intake of tamoxifen is necessary for improved long-term survival, but noncompliance compromises survival.

## PATIENT COMPLIANCE

Tamoxifen’s effectiveness is well documented, but it is not without its concerns. There are safety and efficacy issues when taking tamoxifen with other medications. Patient education reduces complications and improves tamoxifen’s effectiveness, resulting in improved long-term survival. The patient teaching sheet, "Precautions for Patients Taking Tamoxifen" (see Appendix A), serves as a reference for patients and providers on which medications and supplements to avoid while taking tamoxifen.

Patient education is mandatory for medication adherence and the improvement of long-term survival. In a thorough literature review, Chlebowski, Kim, and Haque (2014) report that 30% to 80% of women do not take their daily tamoxifen. Multiple strategies may be implemented to increase compliance, including follow-up phone calls, pill containers, reviewing when prescriptions were filled, encouraging taking medication at the same time every day, changing the time of day (morning to night) to reduce side effects, and e-mail or texting reminders. Meeting with oncology providers on a regular basis makes patients accountable, encourages addressing patient concerns, educates patients on new research reports, and supports healthy behaviors.

## ADVERSE EFFECTS

A major factor in adherence to tamoxifen is the adverse effects that patients experience. Patients who felt poorly informed regarding adverse effects with any adjuvant hormonal breast cancer therapy were more likely to stop therapy prematurely (Kahn, Schneider, Malin, Adams, & Epstein, 2007). Hot flashes are the most commonly reported adverse reaction, which is thought to be related to the central nervous system antiestrogenic effects causing thermoregulatory dysfunction (Stearns et al., 2002). Tamoxifen has been shown to be associated with an increased rate of venous thromboembolic events (Early Breast Cancer Trialists’ Collaborative Group, 1998). There is an increased risk of both endometrial cancer and uterine sarcoma with tamoxifen. These results are of statistical significance, but it is noted that 10 years of tamoxifen prevents 30 times as many breast cancer deaths (Azvolinsky, 2013). Also, with early detection, endometrial and uterine cancers can be treated. Endometrial hyperplasia, endometriosis, polyps, and uterine fibroids have occurred, resulting in reports of abnormal bleeding. Liver abnormalities such as fatty liver, cholestasis, and hepatic necrosis have occurred in rare instances. Reports of visual acuity, corneal changes and color perception changes should be promptly investigated. Tamoxifen is associated with other adverse events including nausea, vomiting, weight loss, weight gain, sexual dysfunction, arthralgia hypertension, and lymphedema in premenopausal women. There is a noted decline in bone mineral density, which may be associated with an increased risk of fractures (Watson Laboratories, 2011). Although all of these concerns are valid and documented, appropriate screening and education can reduce complications and patient concerns.

## INTERACTIONS

Many patients are on medications that may interfere with the effectiveness of tamoxifen. Antidepressants such as paroxetine, fluoxetine, bupropion, and duloxetine may reduce the effectiveness of tamoxifen by inhibiting the conversion of tamoxifen to its active metabolites by inhibition of the cytochrome P450 2D6 (Jin et al., 2005; Kelly et al., 2010; Sideras et al., 2010). Neuroleptics such as thioridazine, perphenazine, and pimozide; certain anti-antimicrobials such as terbinafine and quinidine; and other medications like cinacalcet may also reduce the effectiveness of tamoxifen by inhibiting the conversion of tamoxifen to its active metabolites by the inhibition of the cytochrome P450 2D6 (Sideras et al., 2010). Other antimicrobials such as moxifloxacin and ciprofloxacin may impact cardiac function, especially with a preexisting condition such as an arrhythmia (Slovacek, Ansorgova, Macingova, Haman, & Petera, 2008). Cardiac medications should be reviewed with cardiologists, as certain medications may exacerbate heart conditions (Crewe, Ellis, Lennard, & Tucker, 1997; Iusuf et al., 2011; Mizutani et al., 2008; Slovacek et al., 2008; Tenni, Lalich, & Byrne, 1989). Whenever patients start new medications, it is important to remind them to review their current medications with their oncology provider.

## SURGICAL CONSIDERATIONS

Advanced practitioners should discuss holding tamoxifen for several days before and possibly up to 2 weeks following surgery. The combination of surgery and tamoxifen increases the risk of venous thromboembolism. Tamoxifen plasma levels decline with an elimination half-life of 7 to 14 days (Hussain & Kneeshaw, 2012). Additionally, tamoxifen and its metabolite, 4-hydroxytamoxifen, significantly inhibited the ability of platelet aggregation (Johnson et al., 2017). In certain surgical situations, tamoxifen may be held to prevent such complications. Input from both the surgeon and the medical oncologist regarding the risks and benefits should be discussed and an appropriate plan shared with the patient.

Supplements of garlic, ginger, ginkgo, glucosamine, green tea, guarana and/or high doses of vitamin E and fish oil should also be stopped before and after surgery, as they may interfere with platelet function (Ang-Lee, Moss, & Yuan, 2001; Backon, 1991; Benjamin, Muir, Briggs, & Pentland, 2001; Bydlowski, D’Amico, & Chamone, 1991; Bydlowski, Yunker, & Subbiah, 1988; Carden, Good, Carden, & Good, 2002; Chamone et al., 1987; Knudsen & Sokol, 2008; Liede, Haukka, Saxen, & Heinonen, 1998; McMahon & Vargas, 1993; Pizzorno & Murray, 1999; Rosenblatt & Mindel, 1997; Rowin & Lewis, 1996; Sotaniemi, Haapakoski, & Rautio, 1995; Srivastava, 1989; Thomson et al., 2002; Vanschoonbeek et al., 2004; Vuksan et al., 2000).

Some herbs, supplements, and other products can be dangerous for patients taking tamoxifen. Although certain products (see Appendix A) are safe when taken as part of a regular diet, they may cause problems when they are taken as supplements in a concentrated form (Pizzorno & Murray, 1999).

## EDUCATION

Patient education provides patients with some control over their tamoxifen management. Medication management is challenging for patients who don’t have the pharmacologic knowledge to understand drug interaction. Patients trust and rely on their providers to educate, guide and support them through their tamoxifen tenure. Reinforcing verbal education with a resource such as the "Precautions for Patients Taking Tamoxifen" handout provides a quick reference for patients and staff.

**Appendix A:**
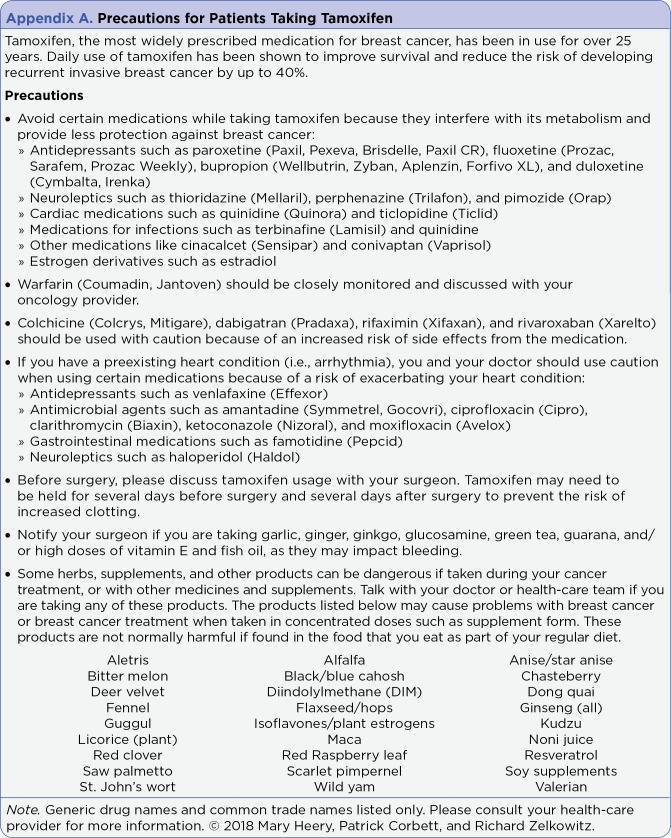
Precautions for Patients Taking Tamoxifen
